# The use of extracorporeal blood purification therapies and sequential extracorporeal support in patients with septic shock (EROICASS): a study protocol for a national, non-interventional, observational multicenter, prospective study

**DOI:** 10.1186/s44158-024-00153-7

**Published:** 2024-02-26

**Authors:** Silvia De Rosa, Fiorenza Ferrari, Massimiliano Greco, Vincenzo Pota, Michele Umbrello, Antonella Cotoia, Laura Pasin, Federico Nalesso, Gianluca Paternoster, Gianluca Villa, Sergio Lassola, Sara Miori, Andrea Sanna, Vicenzo Cantaluppi, Marita Marengo, Fabrizio Valente, Marco Fiorentino, Giuliano Brunori, Giacomo Bellani, Antonino Giarratano

**Affiliations:** 1 Centre for Medical Sciences (CISMed), University of Trento, 38122 Trento, Italy; 2 Anaesthesia and Intensive Care, Santa Chiara Regional Hospital, APSS, Trento, Italy; 3 International Renal Research Institute of Vicenza (IRRV), Vicenza, Italy; 4 Department of Anesthesia, Intensive Care and Emergency, Fondazione IRRCS Ca’ Granda Ospedale Maggiore Policlinico Milan, Milan, Italy; 5 IRCCS Humanitas Clinical and Research Center, Milan, Italy; 6 Department of Woman, Child and General and Specialized Surgery, University of Campania “Luigi Vanvitelli”, Naples, Italy; 7 Section of Resuscitation and Anesthesia, ASST Ovest Milanese, Ospedale Nuovo Di Legnano, Legnano, Milan Italy; 8 Department of Medical and Surgical Sciences, Anesthesia and Intensive Care Unit, University of Foggia, Foggia, Italy; 9 Nephrology, University Hospital, University of Padova, Padua, Italy; 10 Division of Cardiac Resuscitation, Cardiovascular Anesthesia and Intensive Care, San Carlo Hospital, Potenza, Italy; 11 Department of Health Sciences, Intensive Care and Pain Medicine Section of Anesthesia, University of Florence, Florence, Italy; 12 Department of Translational Medicine, Nephrology and Kidney Transplantation Unit, “Maggiore Della Carità” University Hospital, University of Piemonte Orientale (UPO), Novara, Italy; 13 Department of Specialist Medicine, Nephrology and Dialysis Unit, Azienda Sanitaria Locale (ASL) CN1, Cuneo, Italy; 14 Nephrology and Dialysis, Santa Chiara Regional Hospital, APSS, Trento, Italy; 15 Department of Surgical, Oncological, and Oral Science (Di.Chir.On.S.), University of Palermo, Palermo, Italy; 16 Department of Anesthesia, Intensive Care, and Emergency, Policlinico Paolo Giaccone, University of Palermo, Palermo, Italy

**Keywords:** Extracorporeal blood purification therapies, EROICASS, Sepsis, Sepsis-associated AKI, Sequential extracorporeal support

## Abstract

**Background:**

Septic shock, a critical condition characterized by organ failure, presents a substantial mortality risk in intensive care units (ICUs), with the 28-day mortality rate possibly reaching 40%. Conventional management of septic shock typically involves the administration of antibiotics, supportive care for organ dysfunction, and, if necessary, surgical intervention to address the source of infection. In recent decades, extracorporeal blood purification therapies (EBPT) have emerged as potential interventions aimed at modulating the inflammatory response and restoring homeostasis in patients with sepsis. Likewise, sequential extracorporeal therapy in sepsis (SETS) interventions offer comprehensive organ support in the setting of multiple organ dysfunction syndrome (MODS). The EROICASS study will assess and describe the utilization of EBPT in patients with septic shock. Additionally, we will evaluate the potential association between EBPT treatment utilization and 90-day mortality in septic shock cases in Italy.

**Methods:**

The EROICASS study is a national, non-interventional, multicenter observational prospective cohort study. All consecutive patients with septic shock at participating centers will be prospectively enrolled, with data collection extending from intensive care unit (ICU) admission to hospital discharge. Variables including patient demographics, clinical parameters, EBPT/SETS utilization, and outcomes will be recorded using a web-based data capture system. Statistical analyses will encompass descriptive statistics, hypothesis testing, multivariable regression models, and survival analysis to elucidate the associations between EBPT/SETS utilization and patient outcomes.

**Conclusions:**

The EROICASS study provides valuable insights into the utilization and outcomes of EBPT and SETS in septic shock management. Through analysis of usage patterns and clinical data, this study aims to guide treatment decisions and enhance patient care. The implications of these findings may impact clinical guidelines, potentially improving survival rates and patient outcomes in septic shock cases.

## Background

Septic shock, a critical condition characterized by organ failure, presents a substantial mortality risk in intensive care units (ICUs), with the 28-day mortality rate possibly reaching 40% [1]. The conventional management of septic shock typically entails antibiotics, supportive organ care, and surgical interventions as needed. In recent decades, researchers have explored extracorporeal blood purification therapies (EBPT) as supplementary treatments for immunomodulation during septic shock, including methods like high-volume hemofiltration (HVHF), the use of high cut-off (HCO) membranes, adsorption techniques, and plasmapheresis [2]. However, the clinical effectiveness of these EBPT technologies remains a topic of debate, necessitating verification through multicenter randomized controlled trials (RCTs). Septic shock frequently leads to acute kidney injury (AKI), impacting a significant portion of patients afflicted by this condition [3, 4]. Critically ill patients with sepsis-associated AKI who require renal replacement therapy (RRT) face an increased risk of mortality [3, 5, 6]. A novel approach, sequential extracorporeal therapy in sepsis (SETS) [7–9], has been proposed for multiple organ dysfunction syndrome (MODS) arising from endotoxic or septic shock. SETS envisions extracorporeal therapies serving as broad-spectrum support, sequentially replacing or supporting the function of multiple organs such as the heart, kidneys, liver, and lungs. Despite numerous studies, decisions about initiating EBPT or RRT in septic shock patients often rely on local clinical practices and the attending physician’s judgment. The optimal timing, modality, duration, and anticoagulation strategies for these therapies remain unclear. Particularly, in the Italian context, there remains a notable scarcity of high-quality data regarding the utilization of RRT and EBPT. Given this deficiency, this study aims to examine and describe the utilization of EBPT in septic shock patients, investigating any potential association between EBPT utilization and 90-day mortality. The study will also offer insights into EBPT utilization based on the presence of AKI in the context of septic shock in Italy.

### Study aim

The EROICASS study will assess and describe the utilization of EBPT in patients with septic shock. Additionally, we will evaluate the potential association between EBPT treatment utilization and 90-day mortality in septic shock cases in Italy.

## Methods

### Design

The EROICASS study is a national, nonprofit, non-interventional, multicenter observational prospective cohort study. The EROICASS study is co-sponsored by Anesthesia and Intensive Care at Santa Chiara Regional Hospital, APSS Trento, Trento, and the Società Italiana di Anestesia Analgesia Rianimazione e Terapia Intensiva (SIAARTI). The steering committee will grant authorship based on personal involvement, following the Vancouver definitions [10]. A group authorship (“SIAARTI study group”) will be established, encompassing all investigators from the participating centers, in accordance with predefined rules for authorship. The study adheres strictly to the principles outlined in the Helsinki Declaration and complies with all relevant local regulations governing patient ethics and consent. Moreover, the study design and reporting will adhere to the Strengthening the Reporting of Observational Studies in Epidemiology (STROBE) checklist to ensure transparency and completeness in reporting our findings.

### Setting

All consecutive patients with septic shock at the participating centers will be prospectively enrolled. Patient enrollment will coincide with ICU admission, and monitoring along with data recording will continue until hospital discharge. This study is observational in nature; enrolled patients will not receive additional treatments beyond those routinely administered in the ICU. All clinical parameters, including those associated with EBPT treatment, will be documented in the web-based CRF.

#### Study population

##### Inclusion criteria

- Septic shock at ICU admission according to sepsis-3 guidelines11
- Age ≥ 18 years

##### Exclusion criteria

- Lack of consent
- Life expectancy at the time of implementation less than 24 h
- Need for extracorporeal membrane oxygenation (ECMO)

#### Outcome measured

##### Primary outcome

Primary outcome is to assess the proportion of EBPT utilization in septic patients.

##### Secondary outcome

1. To assess association between EBPT and 28-day and 90-day mortality in patients with septic shock.
2. To assess the proportion of EBPT utilization in septic patients, according to the presence/absence of AKI.
3. To describe indication, modality, and duration of EBPT in septic patients, also according to the presence/absence of AKI.\
4. To assess proportion, indication, modality, and duration of SETS in septic patients with renal failure.
5. To assess association between SETS utilization and 90-day mortality in septic patients with renal failure.
6. To describe changes in blood lactate, vasoactive inotropic score (VIS), Sequential Organ Failure Assessment (SOFA) score, and AKI staged (with KDIGO criteria) before and after starting EBPT and SETS.
7. To evaluate relationship between 90-day mortality and changes in blood lactate, VIS, SOFA score, and AKI staged (with KDIGO criteria) in patients undergoing EBPT and SETS.
8. To evaluate relationship between fluid accumulation at EBPT initiation and 90-day mortality.
9. To assess the frailty score change at 28- and 90-day post-discharge.
10. To identify possible risk factors (e.g., baseline clinical parameters, features of EBPT) for 90-day mortality in patients with EBPT or SETS.
11. To investigate the impact of time of EBPT and SETS on renal outcomes, mortality, ICU and hospital length of stay, and 28-day free EBPT (free-SETS days).

### Timeline

**Fig. 1:**
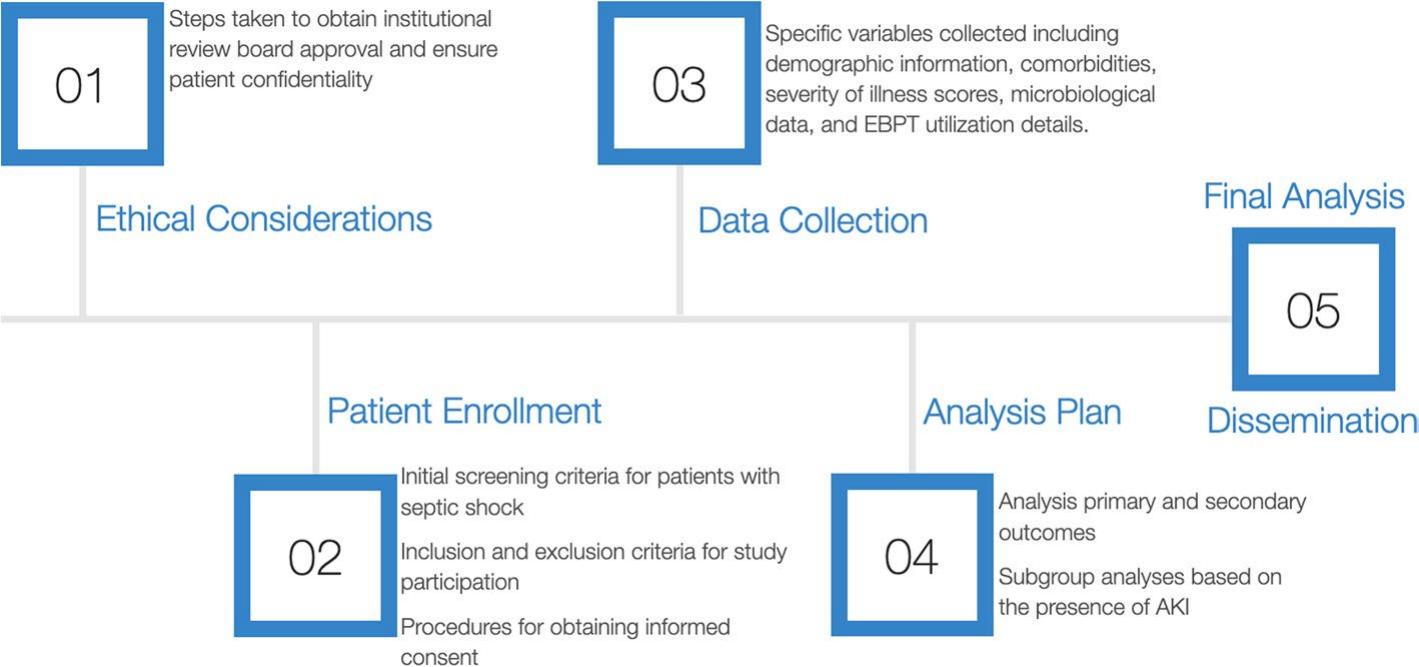
Protocol flowchart for the EROICASS study. The figure presents a flowchart outlining the key steps and procedures followed in the EROICASS study

The study recruits all consecutive patients with septic shock at the participating centers who will be prospectively enrolled (Fig. 1). Patient enrollment will coincide with ICU admission, and monitoring along with data recording will continue until hospital discharge will be prospectively enrolled. The study duration will be 12 months from the first IRB approval. Prior to the initiation of the study in September 2024, we anticipate that a minimum of 30 centers will be prepared for inclusion. It is noteworthy that the study is scheduled to commence before securing the majority of Institutional Review Board (IRB) approvals.

Each participating center will enroll consecutive patients over a 120-day window after the IRB approval.

### Data management

#### Data collection

Variables will be recorded upon ICU admission and monitored over 72 h, extending to ICU and hospital discharge. The following codified data will be collected via a collection form:

- Date, time of ICU admission, and origin
- Demographic data and comorbidities
- APACHE II score, SAPS II score, and SOFA score
- Hemodynamic and perfusion parameters
- Use of vasopressors
- Consciousness assessment (Glasgow Coma Score)
- Blood chemistry tests (platelet count, bilirubin levels, creatinine levels, azotaemia, potassium, sodium, urine quantity)
- Renal function and definition of renal damage according to KDIGO criteria
- Timing, type, and duration of EBPT
- Timing, type, and duration of SETS
- Vital status during ICU and hospital stay
- Long-term outcome post-discharge from ICU

The data will be generated and recorded in participating centers via a web application hosted by SIAARTI using REDCap® software. REDCap provides secure, web-based data capture tools with features including the following:

- Intuitive interface for validated data capture
- Audit trails for tracking data manipulation and export procedures
- Automated export procedures for data downloads to statistical packages
- Data integration and interoperability with external sources
- Data will be recorded in the e-CRF using coding procedures to comply with data protection laws, including EU GDPR. Each patient will be assigned a unique subject ID for pseudonymized data analysis.

Access to REDCap will be granted only to data collecting staff of participating centers, adhering to outlined procedures. Unauthorized access is prevented through hierarchical, role-based access concepts. Data management and retention will be overseen by SIAARTI, ensuring secure storage accessible only to system administrators. Each participating center will have access only to the patient data it generated and recorded on the REDCap platform. Periodic ad hoc reports on collected data will be provided by each participating center. Measures for de facto anonymization will be established during data collection and processing, eliminating the need for name-related patient identification.

### Data management and retention

For data management, the study will utilize the REDCap software [11, 12]. The documentation center will not require name-related identification of individual patients at any point during the study. To ensure de facto anonymization during data collection and processing, all necessary measures will be implemented. SIAARTI will oversee data storage, which will be in a secured, central room accessible only to system administrators. Each participating center will be granted access solely to the patient data it has generated and recorded on the REDCap platform. Additionally, each center will be responsible for providing periodic ad hoc reports on the collected data.

### Statistical analysis

#### Sample size

This is a cohort study aimed at determining the cumulative incidence (absolute risk) of EBPT use in septic shock patients. Since no data are available in the literature, we employed a conservative estimate of 50% as the proportion for EBPT utilization in septic shock patients in the ICU to estimate the study sample size. According to Cochran’s formula, we estimated a sample size of at least 97 patients for estimating EBPT utilization with a 95% confidence level and 10% relative precision. If the number of participating centers is sufficient, the study will enroll a maximum of 385 patients to increase accuracy to within 5%.

### Statistical analysis plan

All parameters will be described by frequencies (absolute and relative) and mean (SD) or median (IQR) based on data distribution. EBPT and SETS will be described in terms of indication, modality, and timing. Differences between patients undergoing and not undergoing EBPT will be assessed using the chi-square test (for discrete variables) or Student’s *T*-test (for continuous variables). Continuous variables not normally distributed will be compared using appropriate nonparametric tests (such as Wilcoxon’s sign test). Similar tests will be conducted to compare patients alive at discharge (from ICU and from hospital) versus those who did not survive, as well as patients with and without AKI (according to KDIGO criteria).

For patients undergoing EBPT or SETS, paired tests (paired *t*-test, Wilcoxon signed-rank test) will be applied to assess statistical differences in lactates, VIS, SOFA score, and AKI stage (based on KDIGO criteria) before and after EBPT or SETS. Multivariable generalized linear regression models will be performed to investigate the association between clinical outcomes (renal outcomes, mortality, ICU and hospital length of stay, free days from EBPT or SETS) and demographic/clinical variables in patients undergoing EBPT or SETS. Depending on the number of participating centers, the characteristics of the centers collected in the case report form may be included in the list of predictor variables for the models.

Relationships will also be analyzed using survival analysis (Kaplan–Meier approach and Cox proportional hazard models) to account for failure time and censoring data. EBPT utilization will be reported in terms of absolute risk and 95% confidence levels. Absolute risk will also be estimated for patients with and without AKI. Estimates will be used to assess the risk ratio for mortality (at 28 days and 90 days) in the entire population and stratified according to the presence or absence of AKI. The same approach will be used to assess SETS utilization in the ICU. All hypothesis tests will be two-sided, and *P* < 0.05 will be considered to indicate statistical significance.

## Discussion

The utilization of EBPT and SETS presents a promising approach for managing septic shock by targeting the inflammatory cascade associated with sepsis and providing comprehensive organ support in cases of MODS. Despite advancements in critical care, sepsis remains a leading cause of morbidity and mortality globally, underscoring the urgency for novel therapeutic interventions [7].

The EROICASS study is dedicated to investigating the utilization patterns and clinical outcomes associated with EBPT and SETS in patients experiencing septic shock. Considering the evolving landscape of sepsis management, there exists a critical need to elucidate the effectiveness and safety profiles of these therapies in real-world settings [13]. Through prospective data collection across diverse medical centers, the study aims to provide valuable insights into the practical application of EBPT and SETS, shedding light on their indications, modalities, timing, and associated outcomes.

Current literature highlights the potential benefits of EBPT and SETS in mitigating the deleterious effects of sepsis, yet substantial gaps persist in understanding their optimal utilization and impact on patient outcomes [14]. The EROICASS study seeks to bridge these gaps by rigorously evaluating the clinical utility of these therapeutic modalities, thereby providing clinicians with evidence-based guidance for therapeutic decision-making.

By elucidating the role of EBPT and SETS in the context of sepsis management, the EROICASS study has the potential to significantly impact clinical practice. Through meticulous data collection and rigorous statistical analysis, the study endeavors to advance our understanding of these therapeutic modalities and their implications for patient outcomes. Moreover, by addressing key questions regarding indications, modalities, and outcomes, the study aims to enhance the quality of care delivered to critically ill patients, ultimately contributing to improved survival rates and better patient outcomes in the challenging landscape of sepsis management.

The comprehensive nature of the EROICASS study underscores its commitment to advancing the field of sepsis management through evidence-based research. By synthesizing current knowledge with new insights gleaned from real-world data, the study seeks to optimize patient care and improve clinical outcomes in this critical area of healthcare.

## Data Availability

Not applicable.
